# Impact of air pollution on hospital admissions in Southwestern Ontario, Canada: Generating hypotheses in sentinel high-exposure places

**DOI:** 10.1186/1476-069X-6-18

**Published:** 2007-07-05

**Authors:** Karen Y Fung, Isaac N Luginaah, Kevin M Gorey

**Affiliations:** 1 Department of Mathematics & Statistics, University of Windsor, Windsor, Ontario, N9B 3P4 and McLaughlin Centre, Institute of Population Health, University of Ottawa, Ottawa, Ontario K1N 6N5, Canada; 2 Department of Geography, University of Western of Ontario, London, Ontario, N6A 5C2, Canada; 3 School of Social Work, University of Windsor, Windsor, Ontario, N9B 3P4, Canada

## Abstract

**Background:**

Southwestern Ontario (SWO) in Canada has been known as a 'hot spot' in terms of environmental exposure and potential effects. We chose to study 3 major cities in SWO in this paper. We compared age-standardized hospital admission ratios of Sarnia and Windsor to London, and to generate hypotheses about potential pollutant-induced health effects in the 'Chemical Valley', Sarnia.

**Methods:**

The number of daily hospital admissions was obtained from all hospitals in London, Windsor and Sarnia from January 1, 1996 to December 31, 2000. We used indirect age adjustment method to obtain standardized admissions ratios for males and females and we chose London as the reference population. This process of adjustment was to apply the age-specific admission rates of London to the population of Sarnia and Windsor in order to yield expected admissions. The observed number of admissions was then compared to the expected admissions in terms of a ratio. These standardized admissions ratios and their corresponding confidence intervals were calculated for Sarnia and Windsor.

**Results:**

Our findings showed that Sarnia and Windsor had significantly higher age-adjusted hospital admissions rates compared to London. This finding was true for all admissions, and especially pronounced for cardiovascular and respiratory admissions. For example, in 1996, the observed number of admissions in Sarnia was 3.11 (CI: 2.80, 3.44) times for females and 2.83 (CI: 2.54, 3.14) times for males as would be expected by using London's admission rates.

**Conclusion:**

Since hospital admissions rates were significantly higher in 'Chemical Valley' as compared to both London and Windsor, we hypothesize that these higher rates are pollution related. A critical look at the way ambient air quality and other pollutants are monitored in this area is warranted. Further epidemiological research is needed to verify our preliminary indications of harmful effects in people living in 'Chemical Valley'.

## Background

In Canada, several reports have been published linking environmental pollution to adverse population health in various cities [1-9]. These reports have consistently shown that Southwestern Ontario (SWO) is second to none as a 'hot spot' in terms of environmental exposure and potential effects [8-11]. This is because the region is very industrialized and communities in this region are exposed to repeated episodes of short-term and long-range transport of air pollutants [5,8,12]. In this area, transported air pollution is characterized by low levels of primary gaseous pollutants (SO_2 _and NO_2_) and moderately elevated concentrations of particles and ozone [5]. According to these authors, episodes of elevated sulfate and ozone concentrations occur frequently throughout the region, especially during the summer and early fall. Many environmental pollutants such as particulate pollutants resulting from the heavy traffic on highways in the region are generated both locally and regionally, and can be carried a long way by winds, affecting areas far removed from the source of the pollution. Also, data analyses strongly indicate that neighboring US states (Ohio, Illinois and Michigan) are significant contributors to elevated levels of ozones and inhaled particles in the region (Ontario Ministry of the Environment (MOE) [13].

**Figure 1:**
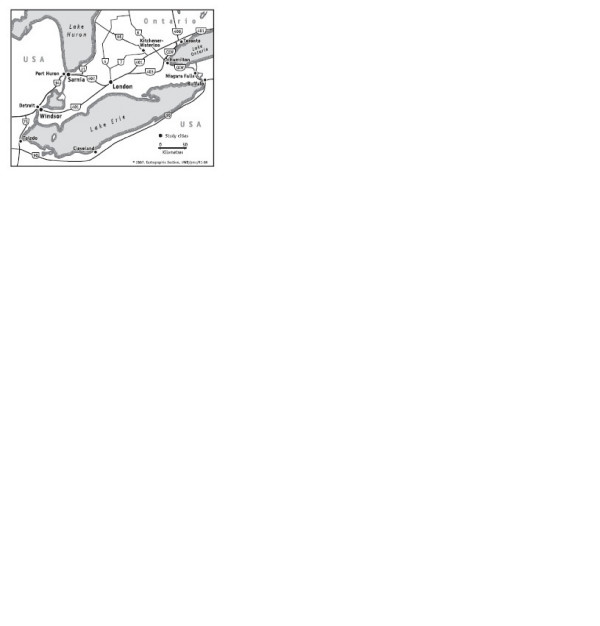
Map of Southwest Ontario, Canada.

There are three major cities in SWO: London, Windsor and Sarnia (Figure 1). The city of London (42° 59' 00" N – 81° 14' 00" W) is located along Highway 401. The city is noted for its economic diversity and is the home of many branches of industry, corporate offices, medical and educational facilities. It is also a manufacturing, distribution and financial center. Using 1981–1991 data, Burnett et al. [8], found that London has the third highest (10%) increased risk of death attributable to change in mean air pollution in 11 Canadian cities. Nevertheless, as compared to Windsor and Sarnia, London is usually referred to as a clean city.

The city of Windsor is located 42° 18'N, 83° 01'W. The city has major industries that include three automobile assembly plants, an engine plant and a foundry, and a scrapmetal recycling plant. In addition to the outstanding problem of transboundary air and water pollution from Ohio, Illinois and, Michigan, the city is immediately downwind of major steel mills with associated coking operations in Detroit; the wastewater treatment plant of the city of Detroit and associated sludge incineration facilities, and a major power plant which until recently was coal fired. Consequently, Windsor and the surrounding communities have been identified as an "Area of Concern" (AOC), and is in need of further health investigation [9]. A community-health profile of Windsor by Gilbertson and Brophy, suggested 'alarming trends' of mortality and morbidity higher than the rest of the province of Ontario [10]. This work aroused a lot of public sentiments and several calls were made for further investigation in what may be happening in the entire region.

Sarnia is located (42° 18'N, 83° 01'W). The city is often referred to as 'Chemical Valley', named because it is a centre for more than 40% of Canada's chemical industry, with major companies like Bayer, Dow Canada, Nova Chemicals and Suncor all having plants located there. These industries are all clustered along the St. Clair River. This area is also the home of Safety-Kleen, one of the largest landfill sites in Canada. With the large number of chemical industry, residents are exposed to a variety of pollutants at work and home. Sarnia is located within the St. Clair River AOC. Three of the major industrial facilities located in Sarnia together contribute more than 16 % of the over 605 million kilograms of suspected respiratory toxicants released by the Top 10 Ontario facilities. In addition to air pollution linked to smog and asthma, other pollutants are released into the air that may affect the health of children and adults and the environment, including lead, mercury, benzene and nickel. Some, like lead and mercury, can be harmful to children's development. Others, like nickel and benzene, are associated with cancer [14].

Gilbertson reported higher rates of hospitalization for cerebral palsy in males in the Great Lakes communities and suggested this as a useful and reliable indicator of community exposures to methylmercury [15]. Mercury is one of the prototypical persistent toxic substances, along with the organochlorine compounds, that have been the central concern of environmental scientists working on the Great Lakes Water Quality Agreement for more than 30 years [16]. Further concerns about health problems in the 'Chemical Valley' were raised when a recent study in a first Nations Reserve indicated a declining sex ratio (2:1) in favour of girls [17]. This drew a lot of media attention and public worry not only in the reserve, but in Sarnia at large.

Both Windsor and Sarnia are located at major Canada-US border crossing points. The terrorist attack of September 11, 2001 (9/11) also brought additional concerns about the effects of air pollution in the region, especially along Highways 401 and 402 transportation corridors. With more vigorous security policies across the US/Canada border-crossing points, there have been increasing delays resulting in long lines of trucks on the streets and highways [18]. The idling trucks are spewing toxic pollutants from their archaic exhaust systems into the air, and prevailing wind carries these pollutants to many areas in the region, further subjecting the entire region to more ambient pollution.

As part of a larger study to investigate environmental health effects and their potential determinants in the region, the aim of this paper is to compare the age-standardized hospital admission ratios of Sarnia, Windsor and London, to generate hypotheses about the adverse health effects of pollution in such highly exposed places as 'Chemical Valley'.

## Methods

Hospital admission records for Ontario Health Insurance Plan (OHIP) patients were obtained from the Canadian Institute for Health Information's (CIHI) Discharge Abstract Database (DAD) for all the hospitals in Sarnia, Windsor and London, Ontario between January 1, 1996 and December 31, 2000. All hospital admissions, admissions with a primary diagnosis of respiratory diseases (based on ICD-9 codes of 460–519), and cardiovascular diseases (ICD-9 codes of 428, 410–414, 427) were analyzed.

Admissions rates for males and females were indirectly age-adjusted. The city of London was chosen as the standard for adjustment because it is the largest of the three cities under study and is considered as the 'cleanest' city. The process of indirect adjustment involves applying the age-specific admission rates of the standard population to a population of interest (Sarnia or Windsor) to yield a number of 'expected' admissions. This process is equivalent to asking, what would be the number of admissions in Sarnia (or Windsor) if people in Sarnia (Windsor) were hospitalized at the same age-specific rate as people in London. The standardized admissions ratio (SAR) of Sarnia (or Windsor) is calculated by the formula [19]:

SAR=total observed admissions in Sarniatotal exp⁡ected admissions in Sarnia calculated from the rates of London=∑ipiania∑ipibnia
 MathType@MTEF@5@5@+=feaafiart1ev1aaatCvAUfKttLearuWrP9MDH5MBPbIqV92AaeXatLxBI9gBaebbnrfifHhDYfgasaacH8akY=wiFfYdH8Gipec8Eeeu0xXdbba9frFj0=OqFfea0dXdd9vqai=hGuQ8kuc9pgc9s8qqaq=dirpe0xb9q8qiLsFr0=vr0=vr0dc8meaabaqaciaacaGaaeqabaqabeGadaaakeaafaqadeGabaaabaGaee4uamLaeeyqaeKaeeOuaiLaeyypa0ZaaSaaaeaacqWG0baDcqWGVbWBcqWG0baDcqWGHbqycqWGSbaBcqqGGaaicqWGVbWBcqWGIbGycqWGZbWCcqWGLbqzcqWGYbGCcqWG2bGDcqWGLbqzcqWGKbazcqqGGaaicqWGHbqycqWGKbazcqWGTbqBcqWGPbqAcqWGZbWCcqWGZbWCcqWGPbqAcqWGVbWBcqWGUbGBcqWGZbWCcqqGGaaicqWGPbqAcqWGUbGBcqqGGaaicqWGtbWucqWGHbqycqWGYbGCcqWGUbGBcqWGPbqAcqWGHbqyaeaacqWG0baDcqWGVbWBcqWG0baDcqWGHbqycqWGSbaBcqqGGaaicyGGLbqzcqGG4baEcqGGWbaCcqWGLbqzcqWGJbWycqWG0baDcqWGLbqzcqWGKbazcqqGGaaicqWGHbqycqWGKbazcqWGTbqBcqWGPbqAcqWGZbWCcqWGZbWCcqWGPbqAcqWGVbWBcqWGUbGBcqWGZbWCcqqGGaaicqWGPbqAcqWGUbGBcqqGGaaicqWGtbWucqWGHbqycqWGYbGCcqWGUbGBcqWGPbqAcqWGHbqycqqGGaaicqWGJbWycqWGHbqycqWGSbaBcqWGJbWycqWG1bqDcqWGSbaBcqWGHbqycqWG0baDcqWGLbqzcqWGKbazcqqGGaaicqWGMbGzcqWGYbGCcqWGVbWBcqWGTbqBcqqGGaaicqWG0baDcqWGObaAcqWGLbqzcqqGGaaicqWGYbGCcqWGHbqycqWG0baDcqWGLbqzcqWGZbWCcqqGGaaicqWGVbWBcqWGMbGzcqqGGaaicqWGmbatcqWGVbWBcqWGUbGBcqWGKbazcqWGVbWBcqWGUbGBaaaabaGaeyypa0ZaaSaaaeaadaaeqbqaaiabdchaWnaaDaaaleaacqWGPbqAaeaacqWGHbqyaaGccqWGUbGBdaqhaaWcbaGaemyAaKgabaGaemyyaegaaaqaaiabdMgaPbqab0GaeyyeIuoaaOqaamaaqafabaGaemiCaa3aa0baaSqaaiabdMgaPbqaaiabdkgaIbaakiabd6gaUnaaDaaaleaacqWGPbqAaeaacqWGHbqyaaaabaGaemyAaKgabeqdcqGHris5aaaaaaaaaa@D2C1@

where pia
 MathType@MTEF@5@5@+=feaafiart1ev1aaatCvAUfKttLearuWrP9MDH5MBPbIqV92AaeXatLxBI9gBaebbnrfifHhDYfgasaacH8akY=wiFfYdH8Gipec8Eeeu0xXdbba9frFj0=OqFfea0dXdd9vqai=hGuQ8kuc9pgc9s8qqaq=dirpe0xb9q8qiLsFr0=vr0=vr0dc8meaabaqaciaacaGaaeqabaqabeGadaaakeaacqWGWbaCdaqhaaWcbaGaemyAaKgabaGaemyyaegaaaaa@30E8@ is a age-specific admissions rate in Sarnia for age group i

nia
 MathType@MTEF@5@5@+=feaafiart1ev1aaatCvAUfKttLearuWrP9MDH5MBPbIqV92AaeXatLxBI9gBaebbnrfifHhDYfgasaacH8akY=wiFfYdH8Gipec8Eeeu0xXdbba9frFj0=OqFfea0dXdd9vqai=hGuQ8kuc9pgc9s8qqaq=dirpe0xb9q8qiLsFr0=vr0=vr0dc8meaabaqaciaacaGaaeqabaqabeGadaaakeaacqWGUbGBdaqhaaWcbaGaemyAaKgabaGaemyyaegaaaaa@30E4@ is the population size in Sarnia in age group i

pib
 MathType@MTEF@5@5@+=feaafiart1ev1aaatCvAUfKttLearuWrP9MDH5MBPbIqV92AaeXatLxBI9gBaebbnrfifHhDYfgasaacH8akY=wiFfYdH8Gipec8Eeeu0xXdbba9frFj0=OqFfea0dXdd9vqai=hGuQ8kuc9pgc9s8qqaq=dirpe0xb9q8qiLsFr0=vr0=vr0dc8meaabaqaciaacaGaaeqabaqabeGadaaakeaacqWGWbaCdaqhaaWcbaGaemyAaKgabaGaemOyaigaaaaa@30EA@ is the age-specific admissions rate in London for age group i.

The following age categories were used in the adjustment: 0–4, 5–14, 15–19, 20–24, 25–54, 55–64, 65–74, ≥ 75 years.

If the SAR were greater than 1, it would mean that more admissions were observed in Sarnia than would be expected based on rates from London.

The lower and upper limits of the 95% confidence interval [19] were:

lower limit=(1−O)2/E
 MathType@MTEF@5@5@+=feaafiart1ev1aaatCvAUfKttLearuWrP9MDH5MBPbIqV92AaeXatLxBI9gBaebbnrfifHhDYfgasaacH8akY=wiFfYdH8Gipec8Eeeu0xXdbba9frFj0=OqFfea0dXdd9vqai=hGuQ8kuc9pgc9s8qqaq=dirpe0xb9q8qiLsFr0=vr0=vr0dc8meaabaqaciaacaGaaeqabaqabeGadaaakeaacqqGSbaBcqqGVbWBcqqG3bWDcqqGLbqzcqqGYbGCcqqGGaaicqqGSbaBcqqGPbqAcqqGTbqBcqqGPbqAcqqG0baDcqGH9aqpdaWcgaqaaiabcIcaOiabigdaXiabgkHiTmaakaaabaGaem4ta8ealeqaaOGaeiykaKYaaWbaaSqabeaacqaIYaGmaaaakeaacqWGfbqraaaaaa@437C@

upper limit=(1+O+1)2/E
 MathType@MTEF@5@5@+=feaafiart1ev1aaatCvAUfKttLearuWrP9MDH5MBPbIqV92AaeXatLxBI9gBaebbnrfifHhDYfgasaacH8akY=wiFfYdH8Gipec8Eeeu0xXdbba9frFj0=OqFfea0dXdd9vqai=hGuQ8kuc9pgc9s8qqaq=dirpe0xb9q8qiLsFr0=vr0=vr0dc8meaabaqaciaacaGaaeqabaqabeGadaaakeaacqqG1bqDcqqGWbaCcqqGWbaCcqqGLbqzcqqGYbGCcqqGGaaicqqGSbaBcqqGPbqAcqqGTbqBcqqGPbqAcqqG0baDcqGH9aqpdaWcgaqaaiabcIcaOiabigdaXiabgUcaRmaakaaabaGaem4ta8Kaey4kaSIaeGymaedaleqaaOGaeiykaKYaaWbaaSqabeaacqaIYaGmaaaakeaacqWGfbqraaaaaa@4549@

where O = observed number of admissions in Sarnia (or Windsor), numerator of SAR

E = expected number of admissions in Sarnia (or Windsor), denominator of SAR.

## Results

The population size, number of all hospital admissions, admissions due to cardiovascular diseases and respiratory disease admissions for males and females in the three cities from 1996 to 2000 are presented in Table 1. Crude rates can be calculated by dividing the number of admissions by the corresponding population size in a given year.

**Table 1:** Population size, number of hospital admissions for London, Windsor and Sarnia from 1996 to 2000.

Gender	City		1996	1997	1998	1999	2000
Male	London	Pop. size	192750	196046	199342	202638	205934
		All adm.	4884	4424	4345	4734	4482
		Cardio.	1636	1520	1475	1666	1491
		Resp.	540	554	571	569	556
							
	Windsor	Pop. size	135925	139039	142153	145267	148381
		All adm.	5178	4782	4801	4851	4323
		Cardio.	1599	1634	1556	1588	1496
		Resp.	825	767	782	818	633
							
	Sarnia	Pop. size	41950	42097	42244	42391	42538
		All adm.	1934	1809	1767	1622	1671
		Cardio.	699	678	709	625	671
		Resp.	355	290	280	284	246
							
Female	London	Pop. size	205855	209329	212803	216277	219751
		All adm.	4185	3990	3758	3965	3879
		Cardio.	1198	1204	1064	1100	1124
		Resp.	521	502	571	624	525
							
	Windsor	Pop. size	142755	145481	148207	150933	153659
		All adm.	4802	4569	4539	4458	4367
		Cardio.	1413	1443	1392	1309	1419
		Resp.	769	650	765	809	647
							
	Sarnia	Pop. size	44525	44747	44969	45191	45413
		All adm.	1860	1681	1645	1563	1588
		Cardio.	647	604	564	552	606
		Resp.	368	296	292	236	238

Table 2 presents the age-standardized admission ratios and their 95% confidence intervals for Sarnia and Windsor compared to London. Since all of the SARs are greater than 1.00 here and all of their confidence intervals do not include 1.00, we concluded that both Sarnia and Windsor had significantly higher admissions rates than London for males and females from 1996 to 2000. For all admissions, the observed ratio in Sarnia ranged from 1.47 to 1.90. For cardiovascular and respiratory admissions, the ratios were even more pronounced, reaching over three times the rate of London for female respiratory admissions in 1996.

**Table 2:** Aged-standardized admissions ratio* (and 95% confidence interval) of Windsor and Sarnia compared to London.

Windsor	All Admissions					
	
		1996	1997	1998	1999	2000
		
	Male	1.49 (1.45, 1.54)	1.53 (1.48, 1.57)	1.57 (1.52, 1.61)	1.46 (1.42, 1.50)	1.38 1.34, 1.42)
	Female	1.63 (1.59, 1.68)	1.64 (1.59, 1.69)	1.74 (1.69, 1.79)	1.63 1.58, 1.68)	1.64 (1.59, 1.69)
Sarnia	Male	1.71 (1.60, 1.76)	1.73 (1.65, 1.81)	1.73 (1.65, 1.81)	1.47 (1.40, 1.54)	1.61 (1.53, 1.69)
	Female	1.90 (1.87, 2.05)	1.86 (1.77, 1.95)	1.94 (1.84, 2.03)	1.76 (1.67, 1.85)	1.82 (1.73, 1.91)

All Cardiovascular Admissions						

		1996	1997	1998	1999	2000
		
Windsor	Male	1.37 (1.30, 1.44)	1.51 (1.44, 1.59)	1.50 (1.42, 1.57)	1.36 (1.29, 1.43)	1.44 (1.37, 1.52)
	Female	1.67 (1.58, 1.76)	1.71 (1.62, 1.80)	1.88 (1.78, 1.99)	1.79 (1.64, 1.83)	1.85 (1.76, 1.95)

Sarnia	Male	1.78 (1.65, 1.92)	1.85 (1.71, 2.00)	2.01 (1.86, 2.16)	1.58 (1.45, 1.70)	1.91 (1.77, 2.06)
	Female	2.33 (2.15, 2.52)	2.16 (1.99, 2.34)	2.28 (2.10, 2.48)	2.18 (2.00, 2.37)	2.34 (2.15, 2.53)

All Respiratory Admissions						

		1996	1997	1998	1999	2000
		
Windsor	Male	2.19 (2.05, 2.35)	1.98 (1.85, 2.13)	1.96 (1.83, 2.10)	2.05 (1.92, 2.20)	1.63 (1.50, 1.76)
	Female	2.10 (1.95, 2.25)	1.84 (1.71, 1.99)	1.92 (1.79, 2.06)	1.87 (1.74, 2.00)	1.31 (1.21, 1.41)

Sarnia	Male	2.83 (2.54, 3.14)	2.29 (2.03, 2.57)	2.15 (1.91, 2.42)	2.19 (1.95, 2.47)	1.94 (1.70, 2.19)
	Female	3.11 (2.80, 3.44)	2.60 (2.31, 2.92)	2.27 (2.02, 2.55)	1.69 (1.48, 1.92)	2.02 (1.77, 2.30)

* SAR= observed admissions/expected admissions calculated from age-specific rates of London

The Windsor rates were somewhere between Sarnia and London and were all significantly higher than London. Sarnia had significantly higher admission rates than Windsor in all cases except for three: respiratory admissions for males in 1998; all admissions for males in 1999; and respiratory admissions for both males and females in 1999. Taken together, these findings certainly seem to sound an alarm. Hospitalization rates in this study's sentinel high-exposure city, Sarnia – in Ontario's 'Chemical Valley' – were much higher than in cities with less air pollution exposure.

To investigate if air pollution may be a factor for the high hospitalization rates in Sarnia, we gathered hourly data of some air pollutants from the monitoring stations obtained from the Ontario Ministry of the Environment. Summary statistics for the pollutants SO_2_, NO_2_, O_3_, and CO from January 1, 1996 to December 31, 2000 are displayed in Table 3 for London, Windsor and Sarnia. All the means of the pollutants are below the ambient air quality criteria (AAQC) set by the Ontario Ministry of the Environment in 2000. During this study period of five years, Sarnia had the highest overall means in SO_2 _and O_3_. The SO_2 _concentration in Sarnia exceeded the AAQC at least one time with an overall maximum of 110.17 ppb. Windsor had the highest overall means for NO_2 _and CO. The number of missing values for each data set is also given in Table 3. Sarnia had more missing data values compared to London and Windsor. Furthermore, it was more likely to encounter missing data gaps of seven or more days, making any approximation of the missing data by moving averages or any other methods nearly impossible. The relatively large number of missing values in Sarnia may have affected the means reported here.

**Table 3:** Missing data days and summary statistics of maximum daily pollutant levels in study period.

Variable (unit)		Mean	Standard Deviation	Minimum/24 hr	Maximum/24 hr	AAQC^†^	Number of Missing Days
SO_2 _(ppb)	London	3.35	2.96	0	28.63		12
	Windsor	7.36	5.06	0	37.50	100/24 hr	8
	Sarnia	9.72	12.72	0	110.17		4
							
NO_2 _(ppb)	London	18.10	7.86	0	53		54^a^
	Windsor	23.50	7.59	6	50	100/24 hr	11
	Sarnia	16.85	8.13	0	52		100^c^
							
O_3 _(ppb)	London	23.61	12.50	0	77		7
	Windsor	20.62	12.58	0	82	80/hr	6
	Sarnia	25.35	11.86	0	76		54^d^
							
CO (ppm)	London	0.16	0.30	0	3		9
	Windsor	0.60	0.44	0	3	3/hr	5
	Sarnia	0.27	0.29	0	1.4		87^b^

^†^Ambient Air Quality Criteria (Ontario Ministry of the Environment, 2000).

a Seven or more missing days in a row: 2000 – Jan. 7–31; Feb. 1–10

b Seven or more missing days in a row: 1998 – Dec 2–31; 1999 – Jan 1–31

c Seven or more missing days in a row: 1997 – Oct. 8–16; 1998 – Sept. 24–30; Oct. 1–31; Nov. 1–10

d Seven or more missing days in a row: 2000 – Nov. 8–16

e Seven or more missing days in a row: 1997 – Jan. 17–23

## Discussion

We compared age adjusted admissions ratios for the three major cities in SWO with the aim of generating hypotheses as to what could be happening in these cities. Sarnia generally had the highest overall admission rates, while London had the lowest rates with Windsor in between them. This pattern that may be characterized as an ecological dose-response was specifically pronounced for respiratory hospital admissions.

The higher rates of morbidity in Sarnia point to the need for further investigation especially the role of pollution on hospital admissions in the city and surrounding communities. As indicated earlier, the geographic context of Sarnia which currently hosts 40% of Canada's chemical plants suggests that the city may be highly exposed, thus, facing severe pollution-related health problems.

Despite the fact that London and Windsor showed better results as compared to Sarnia, earlier studies that examined the association of ambient air pollution and hospital admissions in both cities reported some significant relationships between air pollution and cardiovascular and respiratory diseases. In London, Fung et al. reported that current day carbon monoxide and coefficient of haze produced significant percentage increase in daily cardiac admissions of 8.0% and 5.7% for people < 65 years old respectively [20]. Fung et al. also assessed the association between daily ambient air quality and cardiovascular disease hospitalization in Windsor and reported, among other things, that short-term effects of SO_2 _are associated significantly with daily cardiac hospital admissions for people ≥ 65 years of age [11]. The percentage increase in daily admission was 2.6% for current day sulphur dioxide, 4.0% for 2-day mean level, and 5.6% for 3-day mean. Similarly, Luginaah et al., also working in Windsor, reported significant associations between NO_2_, SO_2_, and CO and daily hospital admission of respiratory diseases [21]. In this analysis, both the case-crossover and time-series analysis were performed. For females 0–14 years of age, there was one-day delayed effect of NO_2_(relative risk [RR] = 1.19, case-crossover method), current day SO_2 _(RR = 1.11, time-series), current day, one and two day delayed effects for CO by case-crossover (RR = 1.15, 1.19, 1.22, respectively). The findings from London and Windsor provided policy makers with current risks estimates of cardiovascular and respiratory hospitalizations as a result of poor ambient air quality in the two cities.

Substantial missing data values from the single existing continuous air quality monitoring station in Sarnia (Station ID: 14064) prevented our directly examining the association between air pollution and hospital admissions. For example, there were 100, 87, 54 and 25 missing days of seven or more days in a row for NO_2_, CO, and O_3_, respectively, in the period under study. Hence, we cannot conclude that there is an unequivocal link between the higher rates of hospital admissions and air pollution in Sarnia. However, based on this and other studies in Windsor and London, we hypothesize that the increased admission rates in Sarnia are probably due to high levels of pollution which warrants government intervention.

In February of 2004, the Ontario Governments Environmental SWAT Team (now known as the Sector Compliance Branch) conducted an 11-month inspection sweep of Sarnia's industrial sector with the primary goal of ensuring that all facilities in that region were brought into compliance with environmental legislation (Ontario Ministry of the Environment, 2005). The decision to undertake a sweep of this magnitude came after a number of facilities in Sarnia's industrial sector had, during the previous year, allowed potentially harmful chemicals to spill into the St. Clair River; with two of those spills resulting in the temporary closures of water-intake facilities that supply drinking water to communities downstream (Ontario Ministry of the Environment, 2005). The objective of the deployment of the Environmental SWAT Team was to ensure that the operation of the petrochemical and related facilities was in compliance with all applicable provincial environmental legislative and regulatory requirements. The SWAT team inspected 35 petrochemical and related facilities located in Sarnia and in St. Clair Township. Thirty-four out of 35 facilities inspected during the sweep were found to be in non-compliance with one or more legislative and regulatory requirements. In total, more that 260 instances of non-compliance with environmental legislative and regulatory requirements were identified in the 35 facilities. The magnitude of the negligence found during the sweep further reinforces community concerns about the potential impact of the 'Chemical Valley'. (Ontario Ministry of the Environment, 2005). Hence, a critical look at the way ambient air quality and other pollutants are monitored in this area is warranted.

In a heavily polluted area, only consistent monitoring can lead to studies that would likely point to the right policy options among interested stakeholders. In fact, it is not surprising that at the local level, there seems to be a reluctance to acknowledge environmental exposure and public health issues [9,23]. Further epidemiological research is needed to verify our preliminary indications of harmful effects on people living in the 'Chemical Valley'. To shed more light on this missing link, a community health study is planned with self-reported health status and real time ambient air quality monitoring of several pollutants via a mobile unit.

By revealing the scale and significance of the problem through this regional comparison, we hope to stimulate the local community and industrial authorities into looking at how they can work together with the aim of implementing remedial measures. Government response to the environmental exposure issues and their associated health impacts in the border areas between Canada and the U.S.A. resulted in the unveiling of a joint strategy to improve the border air quality [22]. As part of this program, it may be useful to establish more long-term monitoring stations in this region. Consistent monitoring will not only aid in the implementation of effective pollution control policies, but will also aid environmental risk management and communication with the public regarding both short and long term health effects.

## Conclusion

Since hospital admissions rates were significantly higher in 'Chemical Valley' as compared to both London and Windsor, we hypothesize that these higher rates are pollution related. A critical look at the way ambient air quality and other pollutants are monitored in this area is warranted. Further epidemiological research is needed to verify our preliminary indications of harmful effects in people living in 'Chemical Valley'.

## Competing interests

The author(s) declare that they have no competing interests.

## Authors' contributions

KF and IL conceived the study and involved in the preparation of the manuscript. KG was involved in the interpretation of the results and preparation of the paper. All authors have read and approved the final manuscript.
